# Spermatocytic seminoma at the National Institute of Oncology in Morocco

**DOI:** 10.1186/1756-0500-4-218

**Published:** 2011-06-29

**Authors:** Ghizlane G Raiss, Marwane M Benatiya Andaloussi, Soundouss S Raissouni, Hind H Mrabti, Hassan H Errihani

**Affiliations:** 1 Medical oncology Department, National Institute of Oncology, Rabat, Morocco; 2 Department of Urology, Ibn Sina Hospital, Rabat, Morocco

## Abstract

**Background:**

Spermatocytic seminoma (SS) is a distinct testicular germ cell tumor, representing less than 1% of testicular cancers. The clinical features that distinguish ss from classical seminoma are an older age at presentation and a reduced propensity to metastasize. The aim of our work is to underline the epidemiological, clinical, histological, therapeutical and prognostic features of this tumor.

**Findings:**

A retrospective analysis of patients referred to the national institute of oncology with seminoma, identified from the institutional tumor registry, between January 1996 and February 2009, was performed. Information reviewed included demographics, clinical, pathological staging, surgical management, adjuvant treatment and last follow-up. We studied four cases of spermatocytic seminoma, which represented 1% of testicular tumor and 6,4% of all seminoma treated at our institution during the study period. Median age at diagnosis was 45 years (range: 42-48). Mean delay before consulting was 9 months and the mean tumor size was 13,75 cm (10-18 cm). No patient had a history of maldescended testis. The main clinical complaint was unilateral testis mass with low progression. Pathology showed that tumors had a polymorphic appearance with small, intermediate and large cells. In all cases, the tumor was limited to the testis. immunohistochemical studies showed that tumors were negative for all the classical antibodies tested (LCA, cytokeratins, PLAP, lymphoid markers, CD117). Thoraco-abdomino-pelvic CT scan and tumor markers (AFP and hCG) were normal. All patients were Stage I. Treatment consisted on an orchidectomy associated with adjuvant radiotherapy in one patient. After a median follow-up of 6 years ranging from 2 to 15 years, we did not note any relapse or metastasis.

**Conclusion:**

The diagnosis of spermatocytic seminoma must be considered in all patients aged of more than 50 with testicular tumor. With only three cases of metastatic disease confirmed in the literature, this is a subgroup of patients in whom radiotherapy can safely be omitted.

## Introduction

Spermatocytic seminoma (SS) is an uncommon neoplasm first described by Masson in 1946 and rarely occurs before the fifth decade. It represents 1 to 2% of germ cell tumors and 4 to 7% of all seminoma patients [1-3]. Unlike classical seminoma originated from undifferentiated germ cells, spermatocytic seminoma may derive from spermatogonia and represented a more differentiated type of germ cell neoplasm. To date, more than 200 cases have been reported, most of them with benign behavior [1-5]. Because of its rarity, the diagnosis of spermatocytic seminoma is difficult, posing the problem of differential diagnosis essentially with testicular lymphoma and classical seminoma, especially after middle age. Immunohistochemical staining can be extremely helpful to assess the diagnosis based on the negativity of all tested classic markers [6,7]. Spermatocytic seminoma rarely metastasizes and there is no documented benefit of radiotherapy or preventive chemotherapy [8-10]. The aim of our work is to underline the epidemiological, clinical, histological, therapeutical and prognostic features of these tumors through a retrospective study conducted at the National Institute of Oncology which is considered the largest institute on cancer treatment in our country.

## Patients and Methods

Between 1996 and 2009, 396 patients with testicular neoplasms of which 160 seminoma were seen. Review of these cases revealed 4 cases of spermatocytic seminoma with an incidence of 2.3%. All patients had undergone orchidectomy at other hospitals and had been referred for subsequent management. Data were collected from patient medical files and the following parameters were recorded: age, primary tumor size, presence of lymphovascular invasion in tumor, presence of rete testis invasion, stage of disease at presentation, relapse and survival. All histological diagnoses have been reviewed at our institution where extensive immunochemistry was performed. Characteristics of spermatocytic seminoma, were appreciated on closer inspection with a tripartite cell population composed of a small lymphocyte-like, intermediate-sized and large cells Immunohistochemically, markers including LCA, CD30, CD20, vimentin, PLAP, AFP, EMA, cytokeratin AE1/AE3, and CD117 were tested in the four cases Patients were staged with thoraco-abdomino-pelvic CT scan, tumor markers (alphafetoprotein AFP and beta-human chorionic gonadotrophin hCG) and serum LDH. All patients had were staged according to the American Joint Committee on Cancer (AJCC 2007/TNM staging system). Three patients were managed with inguinal orchidectomy followed by surveillance. In one patient, a scrotal orchydectomy was done initially for scrotal traumatism in other establishment where a tumor was discovered then referred to our institute. Diagnosis of spermatocytic seminoma was done and a resection of the spermatic cord was performed. This patient received prophylactic radiotherapy.

Radiotherapy was delivered using megavoltage photons, to a dose of 25 Gy in 20 fractions over 4 weeks, prescribed to mid-plane. The treatment volume encompassed the para-aortic and ipsilateral pelvic lymph nodes. A recommended schedule of surveillance for stage I seminoma was adopted in our patients : 4-monthly clinical examination, abdominopelvic CT scan, tumour markers and chest X-ray for the first 3 years, then every six months for year 4 to 7 and finally every 12 months Clinical and pathological information concerning these four cases are summarized in (Table 1).

**Table 1:** Spermatocytic seminoma patient characteristics

Case N°	age	Duration of symptoms(month)	Size(cm)or tumor long axis	Laterality	therapy	metastase	Follow up
1	46	3	10 × 8 × 8 cm	Left	Orchidectomy and radiation therapy.	None	A & W 15 years postop

2	42	12	12 cm	Right	Orchidectomy	None	A & W 8 years postop

3	44	60	16,5 cm	right	Orchidectomy	None	A & W 3 years postop

4	48	18	18 **cm(**whole testis affected)	right	Orchidectomy	None	A & W 2 years postop

A & W = Alive and well with no evidence of disease.

## Consent and statement of ethical approval

As the treatment of each patient was decided by the medical staff of the centre, oral consent was obtained from the subjects and was approved by the institutional review boards of the National Institute of Oncology, Cancer Centre in Rabat. This study was approved by the institutional review boards of National Institute of Oncology, in Rabat

## Results

**Figure 1:**
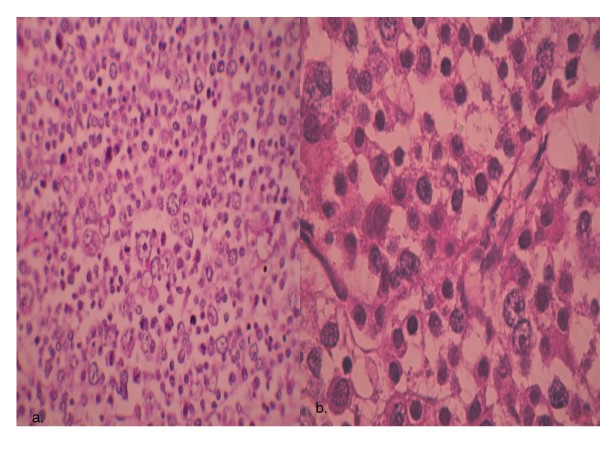
**Spermatocytic seminoma showing a characteristic mixture of small, medium-sized and large and multinucleated cells.(H & Ex200), with round nuclei, and marked mitotic activity (H & E, ×1000)**.

**Figure 2:**
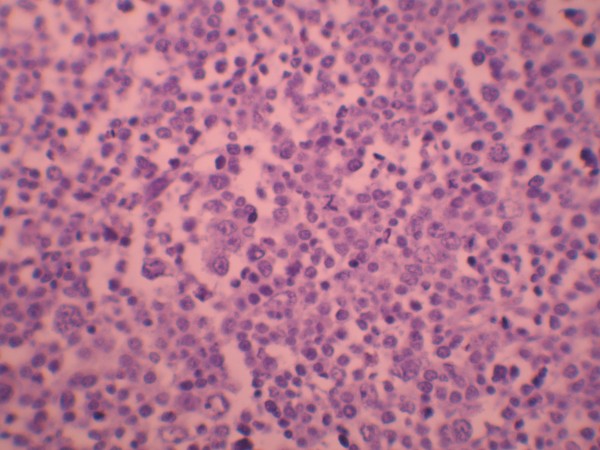
Negativity of tumoral cells for PLAP antibody (×400).

We studied four cases of spermatocytic seminoma, that represents 1% of testicular neoplasm and 2,5% of all seminoma treated at our institution during the study period. Median age at diagnosis was 45 years-old (42-48). All the tumors had arisen in normally descended testes. The main clinical complaint was unilateral non-painful testis mass with low progression. The median duration to consultation was 9 months (range: 3 months to 5 years). The tumor was right-sided in 3 cases and left-sided in one. Serum alphafetoprotein, hCG and LDH were normal. Histologically, all tumors examined showed features of spermatocytic seminoma. Tumors had a polymorphic appearance with small, intermediate and large cells. The stroma displayed variable microcyst formation, without fibrosis or lymphocytic infiltration ( [Figure 1]. None was combined with other germ cell tumors or associated with sarcomatous component. In all cases, the tumor was limited to the testis ( Rete testis and lymphovascular invasion was not observed). In the peritumoral tissue, there was no intratubular germ cell proliferation. Immunohistochemical studies showed that tumors were negative for all the classical antibodies tested (LCA, CD30, CD20, vimentin, PLAP, AFP, EMA, CD117 and cytokeratin AE1/AE3))[Figure 2]. All patients were Stage I (disease confined to the testis and no evidence of metastasis on clinical staging). All patients underwent orchidectomy and only one received adjuvant radiotherapy. The others were placed on surveillance program. After a median follow-up of 6 years ranging from 2 to 15 years, we did not notice any relapse or metastasis.

## Discussion

Spermatocytic seminoma is a rare germ cell tumor, first described by Masson in 1946[11]. It represents a distinct testicular neoplasm with an independent pathogenic pathway and low probability to metastasize. Prognosis is more favorable than classic seminoma in the absence of sarcomatous contingent or metastases. Although rare, these tumors are more common than is suggested by the approximately 200 cases that are currently documented in the literature as case reports and case series. A literature review revealed that the incidence of spermatocytic seminoma in different series varies between 1.7-12% of all serminomas [12], but most of the larger series showed a frequency of 1,1-7,4%[13-16]. For example among 9,658 cases of primary malignant testicular neoplasms recorded in the Australian cancer registries over 20 years, spermatocytic seminoma was identified in 58 cases, yielding an incidence rate of less than 1,1% of all seminoma[15]. Similarly, this uncommon tumor represents at our institution 1,1% of all testicular germ cell tumors.

Phenotypic characterization and origin cell of spermatocytic seminoma (SS) are different from other subtypes of Germ-cell tumours (GCTs). In fact, GCTs are a heterogeneous group of neoplasms characterized by their chromosomal complement and developmental potential [17 = nrc]. GCT characteristics can either be due to the process of tumorigenesis or just a reflection of normal embryonal development, which contributes to the complexity of these tumours [17]. Based on epidemiology, clinical presentation, phenotypic characterization, chromosomal constitution and genomic imprinting, the group of testicular GCTs comprises three of the five GCT entities: the teratomas and yolk-sac tumours of newborn and infants; the seminomatous and non-seminomatous tumours of adolescents and young adults; and the spermatocytic seminomas of the elderly [18,19]. Looijenga and al had presented data demonstrating that spermatocytic seminomas, referred to as the type III GCTs, have a distinct pathogenesis from seminomas and non-seminomas, most likely originating from primary spermatocytes. Genome-wide analyses of genomic changes and expression profiling confirm the origin of spermatocytic seminoma from primary spermatocytes that have at least initiated prophase meiosis. Therefore, the published results indicate that structural chromosomal changes are rare, and that gain of chromosome 9 is the only recurrent imbalance [20]. The paternal pattern of genomic imprinting of spermatocytic seminoma is consistent with this tumour arising from a germ cell that is more mature than a Primordial germ cells [21].

Few spermatocytic seminomas have been studied at the ultrastructural level. Morroni and al have described the ultrastructural features of classical seminoma in comparison with SS. This study indicate that detection of intermitochondrial cement in spermatocytic seminoma confirms a more differentiated phenotype compared with classical seminoma [22].

It is well known that SS is exclusively a testicular tumor, which has never been observed in ovarian or ectopic testis. SS is not associated with any known risk factors for germ cell tumors like cryptorchidism, subfertility or gonadal dysgenesis [13,23].

Clinically, the main difference between spermatocytic and classical seminoma is the age of occurrence. Spermatocytic seminoma tends to occur more commonly, in men aged over 50, while in classical seminoma, the age at diagnosis is between 25 and 40 years. In our series, patients were younger than reported in the literature all patients were aged less than 50. The duration of symptoms was on the whole longer compared with classical seminoma, indicating a slower evolution and less malignant biological behavior. Similarly, in our series, patients' history varied from 3 months to 5 years with an average of 14 months. The large size of the spermatocytic as compared to the classical seminoma was noted by Masson[11]. The size of the tumor was ranged from 10 to 16 cm with an average of 6.6 cm[14], usually replacing the whole testis. Similarly, in the present cases, the mean tumor size was 13,5 cm. The histological characteristics of all the tumors studied were basically similar and conformed to those described by other investigators [9,14]. Spermatocytic seminoma is morphologically characterized by the presence of small, intermediate and large cells with similarities to spermatogonia and spermatocytes, [17] These views received further support from others studies reporting different histogenesis of SS in comparison with CS and based on analysis of DNA ploidy and immunohistochemical profiles. While SS contains diploid to polyploidy cells as the principal finding, CS is predominantly aneuploid [5]. Differential features between Spermatocytic and Classical Seminoma are presentend in table 2. Immuhistochemically, Placental like alkaline phosphatase (PLAP)-negative cells are a typical feature of SS, whereas CS cells stain positively for PLAP [9,24]. However, focal PLAP positivity has been observed [25]. In general, the SS cells show focal, weak c-Kit positivity. Decaussin reports 7 cases of spermatocytic seminoma in which c-Kit was expressed in all cases[2]. This membranous positivity was focal in 4 cases, very strong, and diffuse in the 3 others. In contrast, none of the 11 SS reported by Stoop expressed c-kit [26]. Other markers such as cytokeratin, neuroendocrin, and lymphoid marquers are reported to be negative [1,2,26]. Thus, c-kit appears as the only immunohistochemical positive marker for this tumor. This positivity does not provide a diagnostic aid, first because it is inconstant depending on the antibodies used, then because the classical seminoma, main differential diagnosis, expresses constantly this protein. Finally, in doubtful cases, including those with differential diagnosis against lymphoma, immunohistochemical analysis can be useful in achieving the correct diagnosis. Similar findings were noted in our cases with negativity of all markers tested.

**Table 2:** Differential Features between Spermatocytic and Classical Seminoma

	Classical seminoma	SS
**incidence**	2%	40%

**Usual age on presentation (yrs)**	20-50	≥ 50 ans

**Occurrence in undescended testis (%)**	8-10%	No documented case

**Site**	Testis only	Testis, ovary, mediastinurn, retroperitoneum and pineal region

**Cell types**	one	3 types: small lymphocyte-like, intermediate-sized and large cells

**lymphocytic infiltration**	Rare ou absent	Present, may be abundant

**DNA ploidy**	Aneuploid	Diploid, Hyperdiploid

**intratubular component**	Typical IGCN(intratubular germ cell neoplasia)	Infiltrating component spermatocytic seminoma

**metastases**	Metastases common	3 cases

**PLAP**	positive	Negative

**prognosis**	Stage depending	Excellent. Better than classical seminoma

PLAP: immunohistochemistry for placental alkaline phosphatase;

Another important histological feature is the presence of the anaplastic variant of spermatocytic seminoma. Only three reports describing six cases of this variant has been noted to date [1,27,28]. This rare variant is characterized by constant features including extensive necrosis, multiple mitotic figures, and vascular and tunical invasion. Despite these worrisome features, the presence of an anaplastic component does not seem to impact the excellent prognosis of spermatocytic seminoma. The malignant potential of SS is very low. Only proven three cases of metastatic spermatocytic seminoma have been described [29-31]. These three patients did not receive adjuvant radiation therapy after orchiectomy. In one case, two cycles of carboplatin monotherapy (400 mg/m2) were administered according to the treatment regimen for classical seminoma at the institution [30]. Metastatic disease has been also reported when spermatocytic seminoma is associated with sarcoma. The sarcomatous component is usually rhabdomyosarcoma or undifferentiated, high-grade sarcoma and it appears that the metastatic disease develops usually from the sarcomatous elements [32] The sarcomatous dedifferentiation in the spermatocytic seminoma was associated in the most reported cases with aggressive behavior and poor outcome [32,33].

When metastatic disease was confirmed, cisplatin-based chemotherapy (PEB) was administered as in classical seminoma. This protocol was poorly effective in reducing the retroperitoneal mass in one case [31]. Although metastatic spread is a rare event, observed metastatic cases indicate the need for meticulous staging and some kind of follow-up in patients with spermatocytic seminoma. Following orchiectomy for clinical stage I testicular classical seminoma, active surveillance, adjuvant limited field radiation therapy (RT), or a short course (one or two cycles) of adjuvant single agent carboplatin all offer an extremely high likelihood of cure. The choice of therapy for an individual patient requires a consideration of the patient's ability to comply with a surveillance regimen as well as acute and delayed complications of adjuvant chemotherapy or adjuvant RT. We generally suggest active surveillance for patients able to comply with an intensive follow-up schedule, because of the decreased risk of late complications and because of the ability to achieve the same overall cure rate when patients who relapse are treated appropriately. Primary tumor size greater than 4 cm and invasion of the rete testis have been identified as independent factors associated with an increased risk of relapse in multivariate analysis [34]. However, surveillance is not contraindicated in men with these features, provided the patient understands that the risk of relapse may exceed 30 percent and that they must adhere rigorously to the surveillance protocol. For patients with clinical stage I seminoma for which active surveillance is not appropriate and for those who want to minimize any risk of relapse, adjuvant chemotherapy with single agent carboplatin is suggested rather than RT. In all cases, there is no unanimity in the therapeutic procedure of SS. It was stated that spermatocytic seminoma is a radiosensitive tumor [35], but no direct evidence for this sensitivity was presented and the usefulness of postoperative radiotherapy was doubted. However, the majority of reported patients in the literatture with SS have received postorchidectomy radiotherapy to the draining lymph node area. Where the seven patients with spermatocytic seminoma managed with surveillance in The series of Chung and al [14], Equal numbers of patients in the series of Pendlebury and al underwent surveillance postorchidectomy as received radiotherapy [16]; In the two series, there has been no relapse. SS rarely metastasizes and there is no documented benefit of a preventive chemotherapy. In our small series, although existence of factor of poor prognosis (size > 4 cm), only the first patient treated in 1996 received adjuvant radiotherapy while the 3 others patients were managed by surveillance. Whilst RT or surveillance are both valid methods of management for stage I classic seminoma, active surveillance in the specific subgroup of SS it is more appropriate. The main benefit of surveillance is that it avoids unnecessary treatment and the associated treatment-related adverse effects.

## Conclusion

SS is a distinct neoplasm both clinically and pathologically from classical seminoma and it differs from the latter especially by its behavior, characterized by an almost complete inability to metastasize with only very few examples described with metastatic behavior. The fact that radiotherapy is not necessary is important in view of the fact that many patients with spermatocytic seminoma are elderly and may be adversely affected by treatment.

## List of abbreviations used

SS: Spermatocytic seminoma; PLAP: Placental alkaline phosphatase; LCA: Leukocyte Common Antigen; EMA: Epithelial Membrane Antigen; RT: Radiotherapy.

## Competing interests

The authors declare that they have no competing interests.

## Authors' contributions

RG, BM, RS participated to the acquisition of data and drafting the Manuscript. HM, HE had revised the manuscript. All authors read and approved the final manuscript.
